# Interventricular membranous septal aneurysm: CT and MR manifestations

**DOI:** 10.1007/s13244-015-0456-3

**Published:** 2015-12-21

**Authors:** Carolina Carcano, Jeffrey P. Kanne, Jacobo Kirsch

**Affiliations:** Department of Radiology, Cleveland Clinic Florida, 2950 Cleveland Clinic Blvd, Weston, Fl 33331 USA; Department of Radiology, Mount Sinai Medical center, 4300 Alton Road, Miami Beach, FL 33141 USA; Department of Radiology, University of Wisconsin School of Medicine and Public Health, 750 Highland Avenue, Madison, WI 53726 USA

**Keywords:** Interventricular membranous septal aneurysm, 64 Multi-slice computed tomography (MSCT), Cardiac malformation, MRI, Cardiac imaging

## Abstract

Advanced cardiac imaging is a valuable method to investigate cardiac malformations. The detection of the interventricular membranous septum has clinical significance due to thrombogenic and arrythmogenic predisposition, as well as a role in obstructing the pulmonary flow. This review describes six clinical presentations in which advanced cardiac imaging has been the tool for evaluation, with special emphasis in CT angiography and cardiac MRI sequences.

*Teaching Points*

• *The interventricular membranous septum can predispose patients to thrombogenic and arrythmogenic events.*

• *Subpulmonic stenosis relates to the protrusion of the aneurysm into the right ventricle*

• *During surgery, ventricular pressures of the opened heart become balanced, making the aneurysm less evident.*

## Introduction

The membranous septum represents the midseptal portion of the interventricular septum. This is a compact fibrous segment anatomically associated with the proximal portions of the great arteries. The absence of myocardium in such regions of high pressure gradients can predispose patients to deformation into an interventricular membranous septal (IVMS) aneurysm [1].

Patients with IVMS aneurysm are often asymptomatic; if symptoms develop they are usually related to an associated complication. The aneurysm-like behaviour of this structure predisposes patients to arrythmogenic and thrombogenic events. Arrhythmias may develop given the close relationship of the IVMS to the descending fibres of the conducting system. IVMS aneurysm may be the uncommon cardiac source of cerebral embolism. In addition, given the position of the IVMS in relation to the pulmonary valve leaflets, an aneurysm may bulge into the right ventricle outflow tract [2].

Transthoracic echocardiography, transoesophageal echocardiography, and cardiac angiography are established methods for diagnosis of IVMS aneurysm. However, advanced cardiac cross-sectional imaging offers more accurate and less invasive information [1, 3]. We review the most common manifestations of IVMS aneurysm and the related anomalies by CT and MR imaging.

## Interventricular membranous septum

**Fig. 1:**
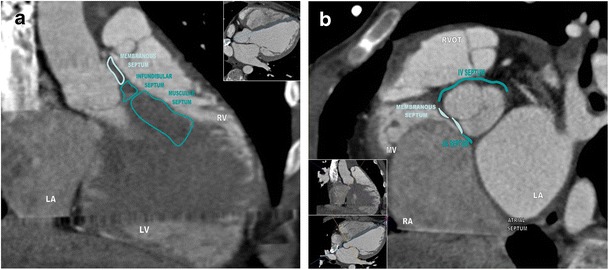
**a** and **b**: Anatomy of the interventricular septum. **a** Parasternal long-axis view of three portions of the interventricular septum: interventricular membranous septum, infundibular septum, and trabecular or muscular septum. **b** Parasternal short-axis view of the cardiac septum: atrialseptum, atrioventricular septum, and the membranous portion of the interventricular septum are shown

Each portion of the ventricular septum has different histological arrangement and relates to different structures: the membranous portion, the inlet, the infundibular, and the muscular septum [4] (Fig. 1a, b).

The membranous portion of the septum is a small structure located below the attachments of the right and non-coronary cusps of the aortic valve that extends to the inlet and the outlet components of the muscular septum [4]. The IVMS is typically an oval or triangular-shaped area of approximately 50 mm^2^, formed by the endocardial lining of the heart chambers and supported only by dense fibrous tissue [5].

The inlet septum corresponds to a lightly trabeculated structure over the inferoposterior portion of the septum, from the atrioventricular valves to their apical annular attachments [6]. The infundibular or distal conal septum is situated between the right and left ventricular outflow tracts [4]. This is a fairly rigid portion of the septum and provides muscular support for the aortic valve, hence septal defects of this segment have been related to progressive aortic insufficiency and aortic outflow obstruction [7]. The muscular or trabecular septum is a heavily trabeculated component of the interventricular septum that extends from the membranous septum to the apex, superiorly to the infundibular septum [4].

## Embryological origin of the IVMS

The ventricular septum starts developing during the 5th week of gestation. The septum grows cephalad as each ventricular chamber enlarges, converging with the ridges of the bulbous cordis and the endocardial cushions. The fibrous portion of the IVMS closes 3 weeks later. Finally, the fused aortopulmonary septum and the fused atrioventricular cushions assemble the muscular portion of the interventricular septum [8, 9].

Congenital ventricular septal defects (VSD) have been described in nearly 100,000 adults and 300,000 children in the United States [10, 11]. They are the most common congenital heart defect after a bicuspid aortic valve [4]. About 80 % of VSDs occur in the membranous septum [11]. Overall, IVMS aneurysm is reported to occur in 0.3 % of patients with congenital heart disease [12].

The IVMS aneurysm is considered to develop idiopathically or related to the spontaneous closure of a VSD after 2 years of age [12]. Other reports relate the development to a previous episode of infection or trauma [2].

**Fig. 2:**
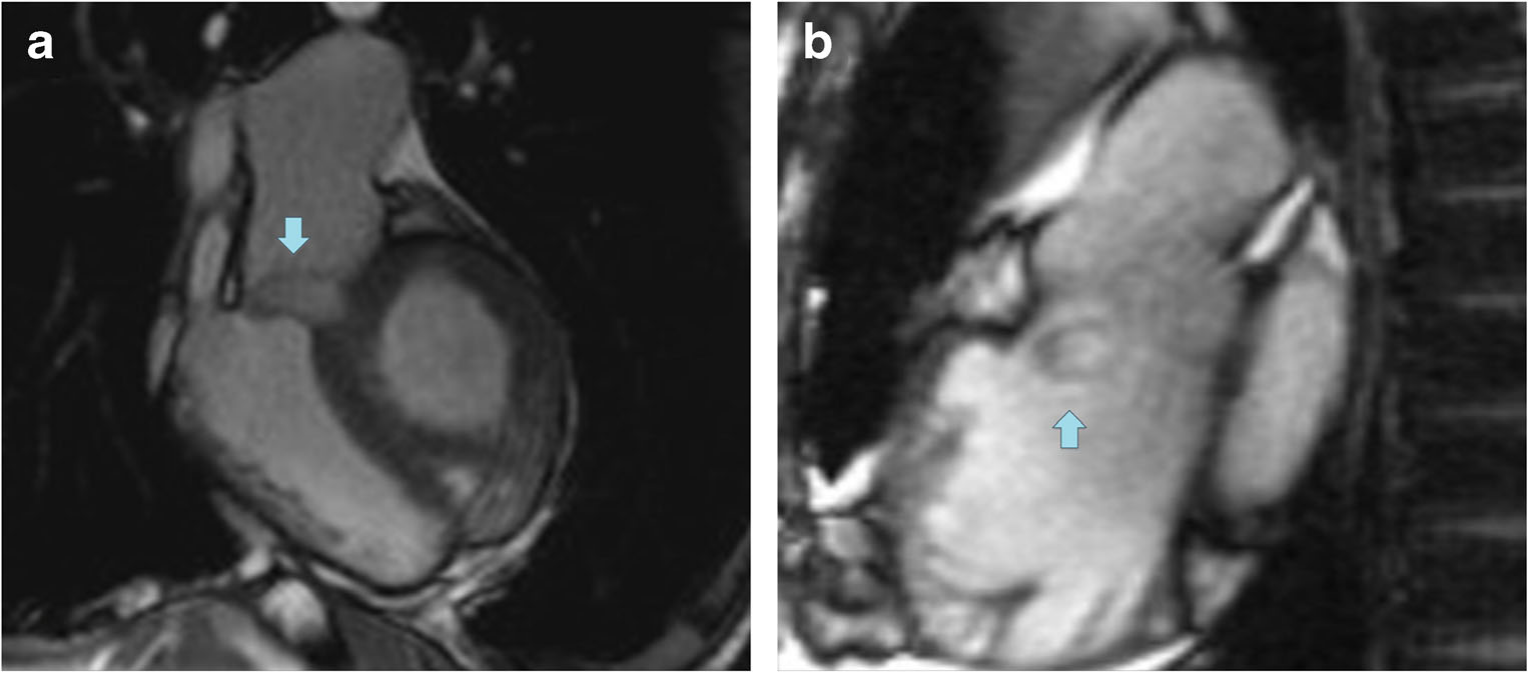
13-year-old boy with corrected TGA and EF 40 %. MRI (bSSFP sequences) in a coronal oblique projection (**a**) shows a finger-like projection of the IVMS (*arrows*) into the RVOT; in the sagittal oblique projection (**b**) through the RV shows the round tip of the IVMS aneurysm in the RV cavity

Rarely, IVMS aneurysm is an isolated anomaly. It is most commonly associated with corrected transposition of the great arteries (TGA) [13]. Allwork et al. found IVMS aneurysms in 25 % of the 32 autopsy specimens of congenitally corrected transposition [13]. In this condition, the membranous septum tends to be larger due to misalignment between the interatrial and interventricular septa. Patients with congenitally corrected TGA and IVMS aneurysm have increased risk of subpulmonic stenosis due to obstructive bulging, among other causes [14] (Fig. 2a, b).

Similarly, IVMS aneurysm has been associated to congenitally abnormal positioning of the aorta. In such cases the course of the vessel is thought to cause a displacement in the septum into a horizontal orientation, thus making it more susceptible to high pressure of the left ventricle [13].

## Clinical presentation

The clinical presentation and the course of disease in IVMS aneurysm relate to the specific morphology of the bulging and the presence of associated defects and complications. The most relevant symptoms are fatigue and exertional dyspnoea, due to severe right ventricular dysfunction, or due to cyanosis related to a right-to-left shunt across a VSD in the aneurysm [15].

A cardiac murmur may be detected and can be accompanied by a thrill [2]. It is attributed to the presence of the aneurysmal sac bulging into the right ventricular outflow tract. However, the intensity or location of the murmur is indistinguishable between patients with IVMS aneurysm and patients with other VSDs [16].

Although some adults with IVMS aneurysm present minimal cardiac enlargement and a systolic click of tricuspid insufficiency, most cases the aneurysm result in no hemodynamic consequence [16]. Occasionally, cases of subpulmonary stenosis have evidence of right axis deviation, right ventricular hypertrophy, and right atrial enlargement on the ECG [4].

## Radiologic technique

Frequently, IVMS aneurysm is an incidental finding in MDCT used for aortic valve surgery planning [17]. MDCT provides high resolution 3D anatomical details of interventricular septal defects, requiring less time and sedation than MR imaging, and also less post-processing time. Low-dose CT protocols minimize radiation exposure [18].

Visualization of IVMS aneurysm is improved with ECG-synchronized MDCT with contrast material in the LV or aorta. Images are obtained with an injection rate of 4–5 mL/s followed by a saline chaser. A bolus-tracking technique is used. These parameters are proposed to opacify the left cardiac chambers and visualize the aneurysm bulging into the unopacified right cardiac chambers. A volumetric data set reconstructed using retrospective ECG-gating techniques at both systolic and diastolic phases, allows multiplanar reconstructions in any desired image orientation, including three-dimensional reformatted images that maximize the visualization of septal defects.

IVMS aneurysms are located just below the aortic valve. Choi et al. reported no difference in morphology of IVMS aneurysms among patients with and without conduction abnormalities [12, 18]. This aneurysm is distinguished from a diverticulum because it is surrounded by fibrous tissue instead of muscle [17].

The direction toward which the aneurysm develops depends on the pressure gradient between ventricles. Therefore, in patients with congenitally corrected TGA, a pulmonary artery protocol may prove useful for better evaluation.

Cardiac MR imaging is also a useful and complementary noninvasive technique for morphological and functional assessment and associated cardiac abnormalities. Morphologic evaluation of the septum is performed using T1/T2-weighted double inversion recovery-prepared black blood sequences [19]. Steady-state free precession (SSFP) cine images can demonstrate intermittent bulging [20].

Four-chamber and coronal oblique images through the LVOT offer the greatest visualization of IVMS aneurysms, with rapid filling and emptying of contrast material from the structure and its absence of solid components (Figs. 2 and 4). Perfusion sequences are helpful to detect the movement of contrast material in possible coexistent septal defects. MRI can be used after treatment to assess the integrity of the patch and identify any residual shunt [19].

## Associated complications

AV valve incompetence. The membranous septum and AV valves are embryologically linked; early insults may affect the normal development of both cardiac structures [21]. Patients with IVMS defects have been reported to have malformed or mal-adherent leaflets of the aortic valve [22]. Acquired aortic valve incompetence relates to impingement of the IVMS aneurysm on the septal leaflet of the valve or distortion of its normal anatomy [13]. These changes may facilitate the appearance of a left-ventricle-to-right-atrium shunt and tricuspid regurgitation [15]. Images obtained in planes parallel to the aortic valve are useful for assessing the valve leaflets [23]. Left coronal oblique and left sagittal oblique planes can be used to visualize aortic valve incompetence and IVMS aneurysm in the same image. These planes are also used to reconstruct perpendicular cross-sectional image of aortic valve by applying multiplanar reformations to detect associated mild aortic valve regurgitation [24].

**Fig. 3:**
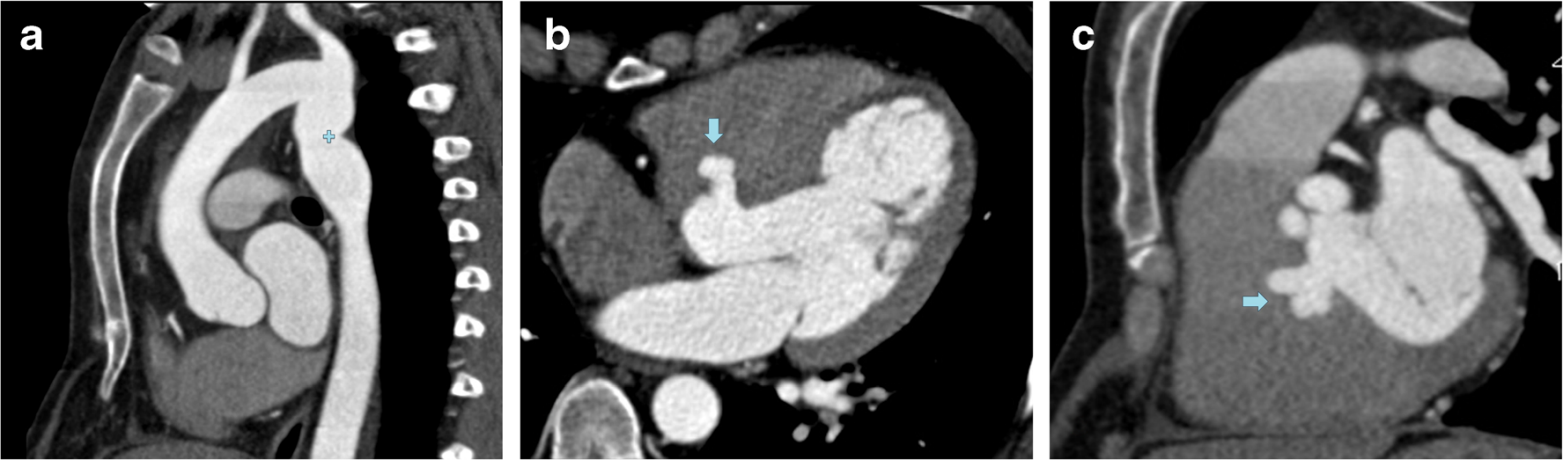
33-year-old man with pectus excavatum deformity and aortic pseudocoarctation. MPR of the aortic arch (**a**) shows an aortic indentation (+) at the level of the proximal descending aorta. Axial (**b**) and coronal oblique MPR (**c**) CT images show a lobulated IVMS aneurysm (*arrows)* projecting into the base of the right ventricle

**Fig. 4:**
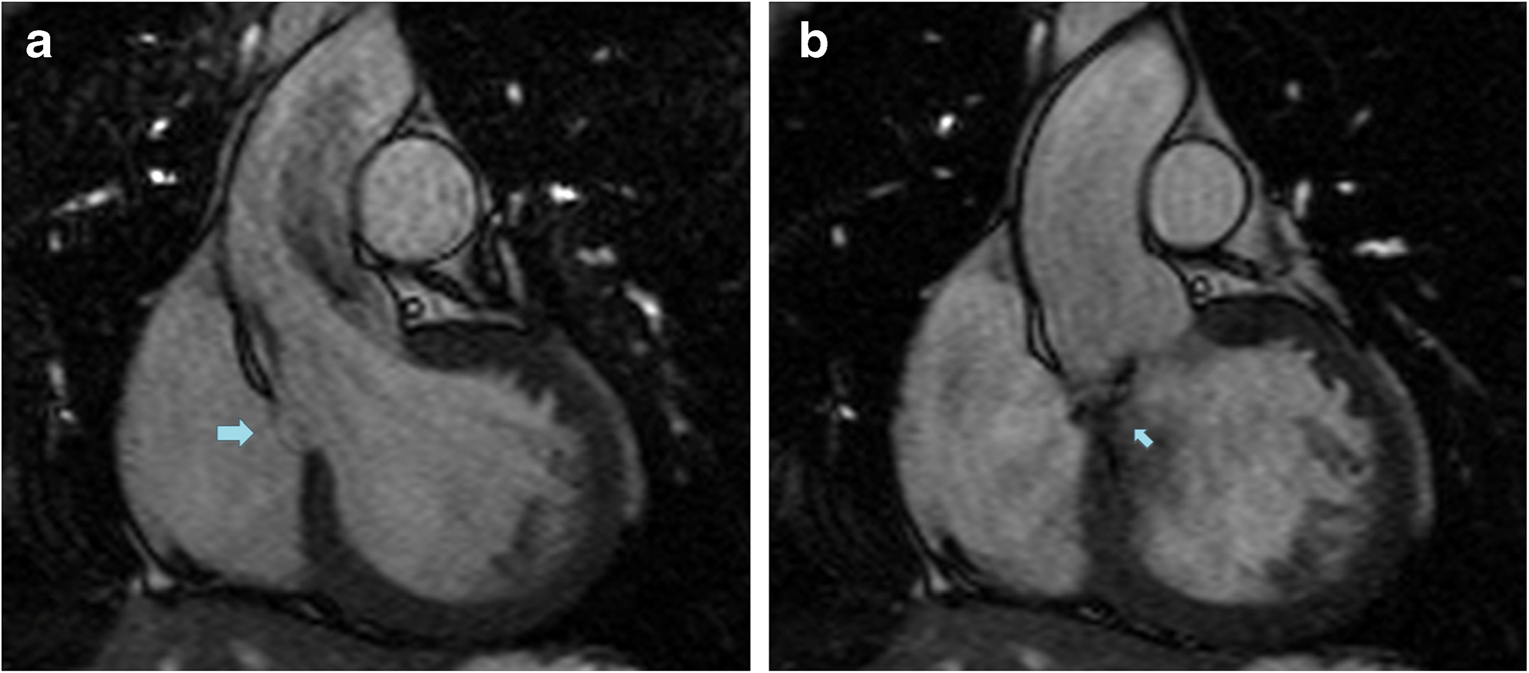
33-year-old man with bicuspid aortic valve and aortic insufficiency. Coronal oblique MRI (bSSFP sequences) through the LVOT shows a small aneursymal out-pouching of the IVMS (*arrow*). Systolic image (**a**) shows dephasing artifact associated with a post-stenotic jet from aortic stenosis in a bicuspid valve, while a diastolic image (**b**) shows an eccentric regurgitant jet (*small arrow*) directed towards the IVMS aneurysm

Aortic insufficiency. The membranous portion of the septum is often in continuity with the right and posterior aortic cusps [5] (Fig. 3a, b, and c). The out-pouching of the IVMS may partially pull the leaflets to prolapse into the aneurysm itself, injuring the supporting apparatus of the valve and causing aortic insufficiency [7]. Aortic valve assessment should include cine imaging created with retrospective ECG gating for visualization of leaflet mobility [23] (Fig. 4a, b).

Given its location, IVMS aneurysm has to be distinguished from an aneurysm of the sinus of Valsalva. Aneurysm of the sinus Valsalva shows saccular dilation of the aortic sinus on cardiac CT, while the IVMS aneurysm lies immediately subaortic [12].

**Fig. 5:**
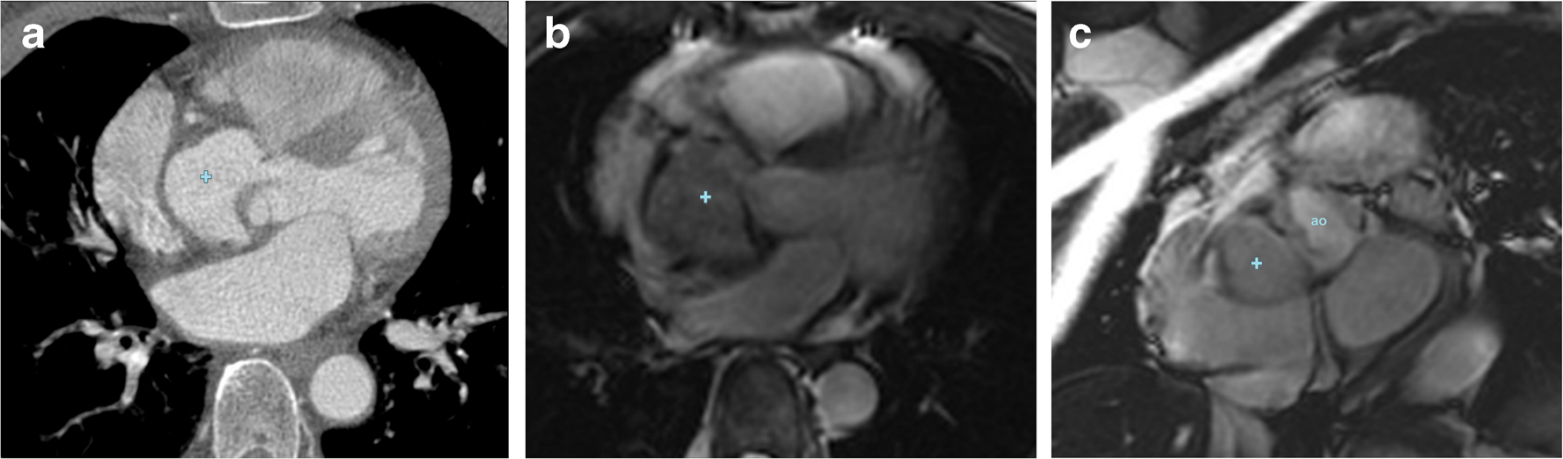
54-year-old man who presented with intermittent syncope. CT axial image MPR (**a**) shows a large IVMS aneurysm (+) bulging and displacing the right ventricle and atrium. MRI (bSSFP sequences) in the axial (**b**) and short axis (**c**) planes reveal a small necked large aneurysm (+) outpouching below the LVOT (ao)

Subpulmonic stenosis. This is the most frequent complication of IVMS aneurysms. The stenosis relates to the protrusion of the aneurysm into the right ventricle or the right atrium. The aneurysm can be imperceptible during ventricular diastole, but become evident when the contraction of the left ventricle pushes the protrusion into the right outflow tract. During surgery, the ventricular pressures of the opened heart balance and the aneurysm can become less evident (Fig. 5a, b, and c).

Arrhythmias and complete atrioventricular block. Anatomically, the membranous septum situates on a very important electric area of the heart. The atrioventricular (AV) node is to be found at the base of the atrial septum, in the Koch triangle. The apex of this triangle is the atrioventricular component of the membranous septum [25]. The fibres of the AV bundle pass along the posterior and inferior margins of the membranous septum [25].

**Fig. 6:**
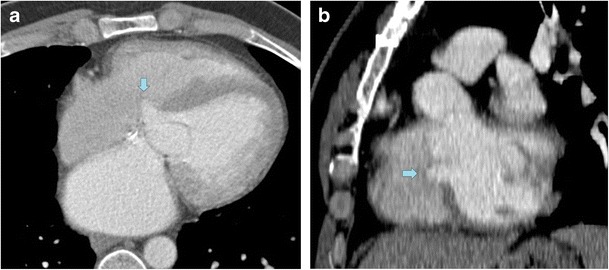
26-year-old man with a history of repaired ASD and atrial arrhythmias. Axial CT image (**a**) and oblique sagittal MPR (**b**) show a small IVMS outpouching into the RV (*arrows*)

**Fig. 7:**
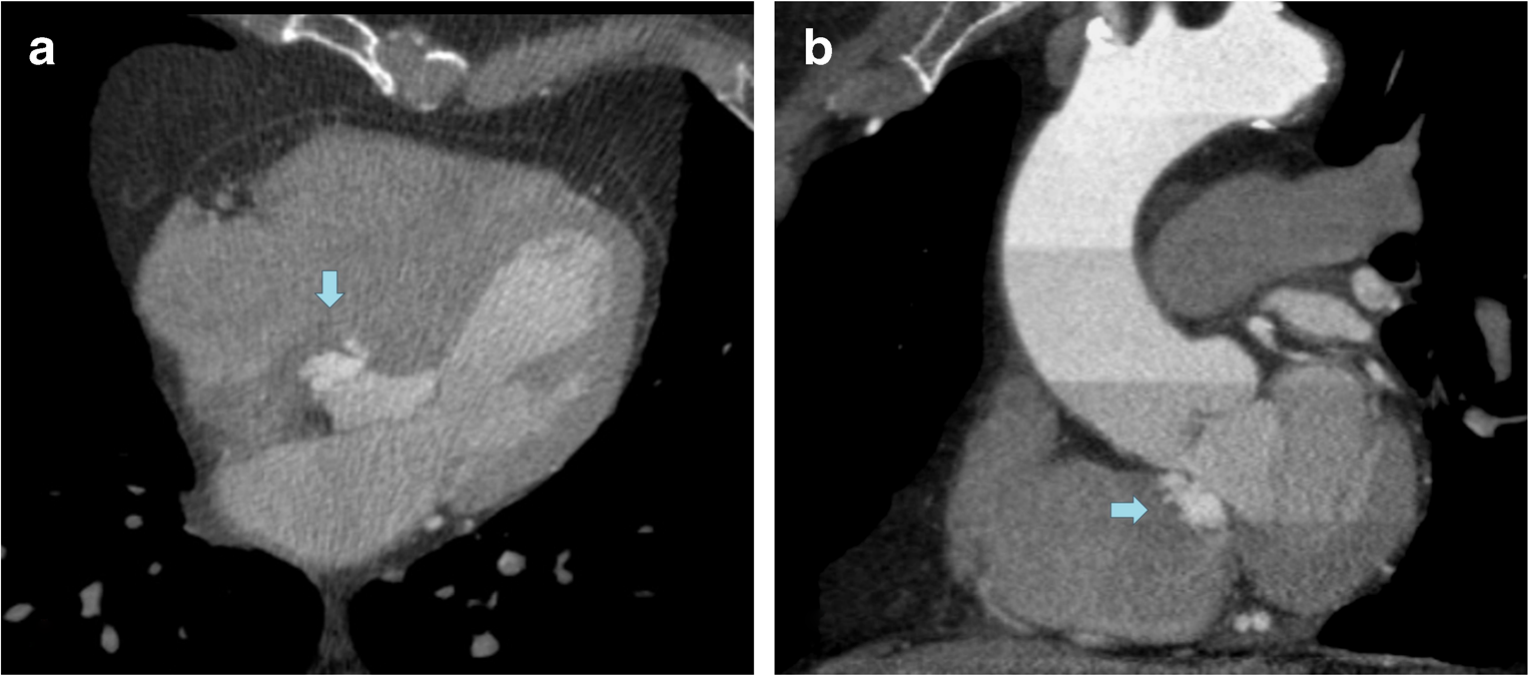
78-year-old man with right heart failure was diagnosed with IVMS aneurysm in the evaluation of cardiac CT. CT axial view and coronal oblique MPR (**a** and **b**) show a small lobulated out-pouching of the membranous portion of the IVMS (*arrows*) extending into the right ventricle

It is postulated the arrythmogenic progression in cases of IVMS aneurysm relates to the stretching of this conducting system at the base of the aneurysm. The relationship with conduction disturbances is supported by the disappearance of the arrhythmia after the aneurysmal repair. In addition, in cases of aneurysmal resection, AV block has developed [13] (Figs. 6a, b and 7).

The most frequent arrhythmias in patients with IVMS aneurysm are ventricular tachycardia, bundle branch block, and AV block [25].

Thromboembolism. The abnormal movement of the ventricular wall in cases of IVMS aneurysm predispose to the turbulent flow implicated in thrombus formation. Some reports have demonstrated echocardiographic finding of thrombus occupying the aneurysmal cavity in patients who had cerebral embolic event [3]. CT density may prove useful in differentiating a thrombus from other tissues [26].

Several studies have demonstrated that contrast-enhanced MR provides the highest sensitivity (87 %) and specificity (99 %) for LV thrombus when compared to TTE and TEE (up to 43 % sensitivity and 96 % specificity), especially in smaller thrombus [27–29]. On gradient-echo cine sequences a thrombus is seen as a low-signal intensity intracavitary mass. However, LV thrombi is best identified immediately after contrast administration, when the homogeneous, strong enhancement of the LV cavity contrasts the dark intraventricular filling defects [30].

Bacterial endocarditis. The high-velocity jet stream created by blood passing through a defect in the aneurysm may allow platelets and fibrin to adhere to the partially denuded surface of endothelium, creating a sterile thrombus, though it can potentially host microorganisms [30].

## Treatment options

Surgical intervention is rarely needed. Repair is mainly indicated when concurrent heart diseases, hemodynamic abnormalities, and aneurysm-related complications are detected.

In the occurrence of a thrombus within an IVMS aneurysm and cerebral embolism, some authors recommend periodic echocardiography checkups. The finding of a mass in the aneurysm suggestive of thrombus, may justify anticoagulation treatment [31]. The aneurysm is recommended to be resected surgically in patients in whom cerebral emboli occur despite anticoagulant therapy, even in absence of echocardiographic evidence of thrombus [31].

Patients with threatening ventricular arrhythmias are potential candidates for direct surgical ablation, which has been shown to have satisfactory results. The ability to localize the cardiac conduction system has allowed improvement in surgical techniques. If the precise location of the conduction system is not properly identified at the operation, complete heart block can develop [30].

Direct suture closure of a VSD with IVMS aneurysm can be unsatisfactory given the residual communication and or recurrence of the aneurysm found postoperatively, as suggested by Yilmaz et al. in his surgical experience with 51 patients. This observation raises the concern for multiple fenestrations overlooked in the base of the aneurysm. Moreover, the portion in the base of the aneurysm weakens and cause further enlargement due to the left ventricle pressure. Closure of the IVMS aneurysm may also cause distortion of tricuspid leaflets [30].

When excision of the aneurysm is recommended, the authors stress the importance of performing concomitant closure of the remaining or possible coexistent septal defect with a patch, anchored with interrupted pledget-buttressed sutures [30].

A large IVMS aneurysm should be operated on during childhood in order to prevent the development of future aneurysm enlargement and the complications associated, more frequently in cases of congenitally corrected TGA. Left unrepaired, a membranous septal aneurysm could cause outflow obstruction with poor postoperative results [13, 30].

## Conclusion

The fibrotic structure of the interventricular membranous septum grounds its predisposition to become aneurysmal. IVMS aneurysm is a rare condition, and in the great majority of cases is not hemodynamically significant itself. However, it can cause numerous complications such as arrhythmias with or without thrombus formation, obstruction of the right ventricular outflow, endocarditis, and valvular insufficiency. MDCT and MRI accurately show IVMS aneurysm and their associated anomalies.
